# Evaluation of concordance between CAD/CAM and clinical positions of abutment shoulder against mucosal margin: an observational study

**DOI:** 10.1186/s12903-018-0534-2

**Published:** 2018-05-02

**Authors:** Jan K. Pietruski, Anna Skurska, Anna Bernaczyk, Robert Milewski, Maria Julia Pietruska, Peter Gehrke, Małgorzata D. Pietruska

**Affiliations:** 1 Private Dental Practice, Białystok, Poland; 2 Department of Periodontal and Oral Mucosa Diseases, Medical University of Białystok, ul. Waszyngtona 13, 15-269 Białystok, Poland; 3 Department of Statistics and Medical Informatics, Medical University of Białystok, ul. Szpitalna 37, 15-295 Białystok, Poland; 4 Private Practice, Ludwigshafen, Germany

**Keywords:** Customized abutment, Abutment shoulder, Soft tissue, Mucosal margin, CAD/CAM technology

## Abstract

**Background:**

While working on CAD/CAM-customized abutments, the use of standard impression copings with a circular diameter produces inconsistency within the emergence profile. It may begin with a collapse of the supra-implant mucosa during impression taking, then lead to a computer-generated mismatch of the position and outline of the abutment shoulder, and consequently result in a compromised outcome of anticipated treatment. The aim of the study was to compare the virtual and clinical positions of the abutment shoulder in relation to the mucosal margin after the abutment delivery.

**Methods:**

Conventional open-tray impression takings followed uncovering surgery. Master casts were scanned with a desktop scanner. Clinical examinations took place after abutment’s insertion and temporization (T1) and prior to cementation of the definitive crown (T2). The distances between the abutment shoulder and marginal soft tissue were measured intraorally in four aspects and juxtaposed with those on the virtual model.

**Results:**

The study evaluated 257 dental implants and CAD/CAM-customized abutments. As T1 and T2 showed, there was a positive correlation between the virtually designed abutment shoulder position and matching clinical location relative to the mucosal margin. In 42.1% of cases, the distance between the mucosal margin and the abutment shoulder did not change. It increased in 36.3% of cases while a decrease occurred in 21.6% of them.

**Conclusions:**

Computer-set position of the abutment shoulder in relation to the mucosal margin can be predictably implemented in clinical practice.

## Background

Due to their numerous advantages, CAD/CAM-customized abutments are widely used in contemporary implant prosthodontics. They enable monitoring of the abutment’s shape, angulation, crown retention and soft tissue contour with an optimal emergence profile [1, 2]. Additionally, positioning of the abutment shoulder slightly below the mucosal margin may prevent a cement-induced peri-implantitis [3–5]. The virtual image of mucosal margin mirrors the tissue outline scanned from the master cast or intraorally [6, 7]. However, the use of standard impression copings with a circular diameter produces inconsistency within the emergence profile. It may begin with a collapse of the supra-implant mucosa during impression taking, then lead to a computer-generated mismatch of the position and outline of the abutment shoulder [8, 9]. An over-contoured transmucosal section of the abutment compresses the peri-implant soft tissue after clinical delivery and may alter its position. Hence, there is a risk of mismatch between the position of the free mucosal margin in a digital image and its actual place.

The importance and scope of digital technology in prosthetics, implant prosthetics in particular, has been growing rapidly. Digital techniques are accepted as additional instruments in diagnostics and prosthetic restoration. The relation between virtual and analogue (intraoral) domains has not yet been fully investigated. The stability of soft tissue is a crucial factor for long-term success in implantology, especially in its aesthetic sector. A number of publications point to complications such as recession, which is a major concern in aesthetically critical regions, where altered soft tissue may expose the titanium abutment unacceptably [10, 11]. When digitally positioning the abutment shoulder, we assume its position to be intraorally identical against the gingival margin, which is often not the case. Since there is no data available on this issue, the aim of the study was to compare the virtual and clinical positions of the abutment shoulder in relation to the mucosal margin after the abutment delivery.

## Methods

### Patients selection

The study was designed as an observational clinical study, with the following inclusion criteria: at least one missing tooth to restore on dental implant, good oral hygiene, no inflammation and a minimum age of 18 years. Patients with general diseases which could influence the healing process, pregnant or feeding women, as well as those for whom implants required additional soft tissue augmentation were excluded. The study was performed in accordance with the Helsinki Declaration of 1975, as revised in 2000. All patients included in this study provided informed written consent for their data to be used for research purposes. We did not seek ethical approval as this was a purely observational study based on routine examination, check-up appointments and patients’ medical notes and such studies are exempt from ethical approval under Polish Law.

All patients underwent thorough hygienic phase – scaling, root planning, oral hygiene instructions, as well as non-surgical and surgical periodontal treatment if necessary. Implants were inserted only after oral hygiene standards were satisfactory (full mouth plaque index ≤20%) and inflammation was under control (full mouth bleeding on probing ≤20%).

### Surgical procedures

The surgical procedures adhered to a two-stage approach. At the second stage (4–8 weeks post-op) the implants were uncovered in a minimally invasive way without sutures. In few cases, where contact of the mucosa with healing abutment was questionable, single interrupted resorbable 5.0 sutures were used. Trans-mucosal healing abutments of 4 mm height 4.5 mm diameter were inserted into implants of diameter 3.5/4.0 while 4 mm height and 5.5 mm into implants of diameter 4.5/5.0 (Dentsply Dental Implants, Mölndal, Sweden). The quality and quantity of the soft tissue surrounding healing abutments were adequate, i.e. the width of keratinized tissue was at least 2 mm on both aspects, buccal and palatal/lingual.

### Prosthetic procedures

Open-tray silicone impressions at implant level were taken 1–1.5 month after implant uncovering. Master cast with silicone gingiva replicas and wax-up-design of the future crown were sent to a commercial CAD/CAM milling center (Atlantis Center Mölndal, Sweden). The mucosal outline and position of each ordered abutment shoulder was consistently designed within the same pattern, respecting four aspects – buccal, palatal/lingual, mesial and distal. The following measures in millimeters were taken into account in designing the position of the abutment shoulder below the virtual mucosa margin:

- in the esthetic region: buccal 2.0 mm, palatal/lingual 0.5 mm, mesial 1.5,mm distal 0.75 mm;
- in the premolar and molar region: buccal 1.5 mm, palatal/lingual 0.5 mm, mesial 1.0 mm and distal 0.5 mm.

**Fig. 1:**
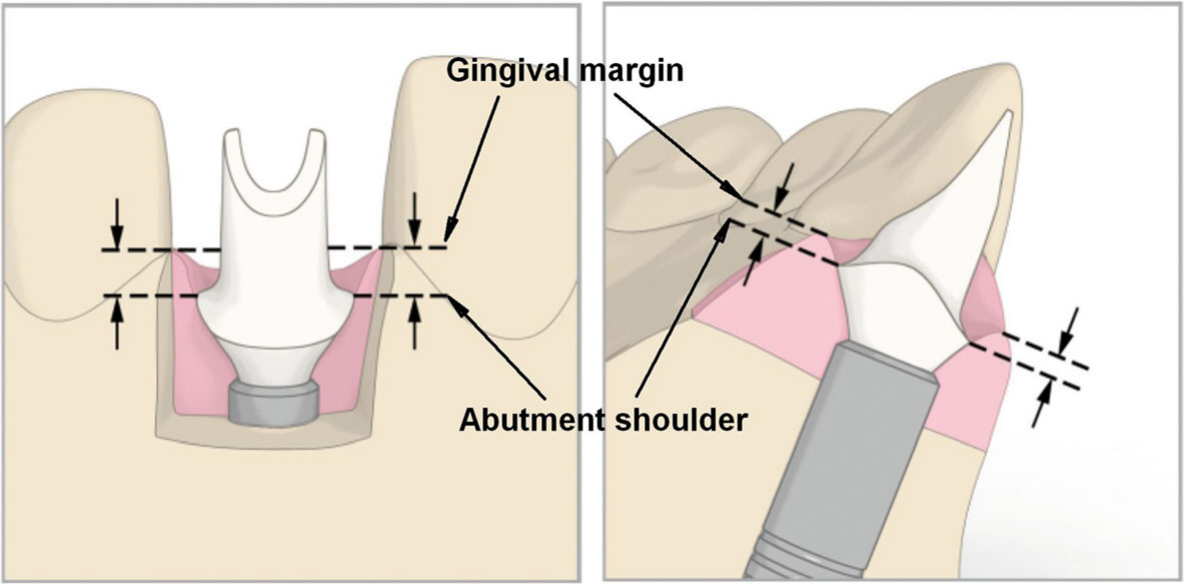
Schematic explaining how to measure shoulder position in accordance to gingival margin (Atlantis™ 3D Editor software, Dentsply Implants, Mölndal, Sweden)

The selected emergence profile was concave and featured a chamfer-margin design (Fig. 1). After being scanned, all abutments were digitally designed using the Atlantis™ 3D Editor software (Dentsply Implants, Mölndal, Sweden) and then milled out of titanium (Atlantis™ Titanium or Atlantis™ Gold Hue, Dentsply Implants, Mölndal, Sweden). After central production the abutments were mounted into implants with an insertion guide. A silicone impression at the abutment level were taken according to the authors’ own procedure described previously [12]. Subsequently, temporary crowns made of composite material (Luxatemp, DMG, Hamburg, Germany) were provisionally cemented in place with Freegenol Temporary Pack cement (GC Dental Products Corp. Toriimatsu-cha, Japan). The definitive crowns, made of monolithic zirconia (Prettau®, ZirkonZahn, Brunico, Italy), were cemented in place at the final appointment with OliSemi Cem (Olident, Krakow, Poland). Cement surplus was thoroughly removed with a dental explorer and floss. X-rays were taken in order to evaluate the crown’s seating on the abutments and check for any cement remnants.

### Clinical measurements

A sole calibrated examiner (AB) carried out two clinical examinations – one directly after the abutment delivery (T1), the other prior to the cementation of the final crown (T2). The distance between the abutment shoulder and free gingival margin (in mm) was measured in four aspects (buccal, palatal/lingual, mesial and distal) with a periodontal probe (PCP UNC 15 (Hu-Friedy, Chicago, IL, USA). All measurements were rounded up to the nearest 0.5 mm.

### Statistical analysis

The measurements from each site (4 points per implant) were used for statistical analysis. The variable evaluated in all tests was the distance between gingival margin and the abutment shoulder. To assess a relationship between qualitative variables, a Chi-Square Test of Independence and Fisher’s exact test were done. Normal distribution was verified by a Kolmogorov-Smirnov test combined with Lillefors amendment and Shapiro-Wilk test. No normal distribution of quantitative variables analyzed was found. Quantitative variables without normal distribution involving two dependent variables were compared in a nonparametric Wilcoxon matched pairs test. A Spearman’s rank order correlation coefficient was also determined. The results were statistically significant at *p* < 0.05. The statistical software was Statistica 12.0 StatSoft (StatSoft. Tulsa, OK, USA).

## Results

The study involved 59 generally healthy individuals aged 26–79 years (42 females, 17 males) and evaluated a total of 257 implants for a single-tooth replacement (164 in the maxilla, 93 in the mandible) with diameters of 3.5, 4.0, 4.5 and 5.0 mm (Osseospeed TXTM, Dentsply Dental Implants, Mölndal, Sweden). There was a positive moderate correlation in terms of distance between the CAD/CAM-designed abutment shoulder and its actual clinical position as relative to the mucosal margin in all implants at both T1 and T2 – total: *R* = 0.37 and *R* = 0.34; locally: maxilla *R* = 0.41 and *R* = 0.35; mandible *R* = 0.31 and *R* = 0.33 respectively (Table 1). There was a strong or very strong positive correlation between virtually designed and actual shoulder’s position at T1 for implants in the anterior zone of the maxilla (*R* = 0.53), and in the mandible (*R* = 0.89) (Table 2). At T2 the correlation for implants in the anterior zone of the maxilla was moderate (*R* = 0.46 and *R* = 0.45) and was very strong for the implants in the anterior region of the mandible (R = 0.89) (Tables 1 and 2. The clinical examination revealed alterations of the mucosal margin against the abutment shoulder. In 42.1% of cases the distance between them did not change. A decrease of vertical dimension of the tissue was noted in 21.6% of all cases while an increase was observed in 36.3% (Table 3). The jaws evaluated separately showed the following results:

- in the maxilla – vertical dimension increased in 34.8%, decreased in 24.1%, did not change in 41.2% of cases
- in the mandible – 39, 17.2 and 43.8% respectively.

**Table 1:** The correlation between the virtual and real position of abutment shoulder in the first (T1) and second (T2) clinical examination

(n)	Correlation between the virtual and real shoulder (T1) (*p-*value)	Correlation between the virtual and real shoulder (T2) (*p-*value)
Total (257)	0.37 (< 0.001)	0.34 (< 0.001)
Maxilla (164)	0.41 (< 0.001)	0.35 (< 0.001)
Mandible (93)	0.31 (< 0.001)	0.33 (< 0.001)
Incisors+canines (35)	0.54 (< 0.001)	0.46 (< 0.001)
Premolars (76)	0.36 (< 0.001)	0.40 (< 0.001)
Molars (146)	0.33 (< 0.001)	0.28 (< 0.001)
ø3.5 (187)	0.38 (< 0.001)	0.36 (< 0.001)
ø4.0 (9)	0.28 (0.09)	0.38 (0.01)
ø4.5 (50)	0.39 (< 0.001)	0.31 (< 0.001)
ø5.0 (11)	0.32 (0.02)	0.3 (0.04)

**Table 2:** The correlation between the virtual and real position of the abutment shoulder in the first (T1) and second (T2) clinical examination depending on the position and diameter

(n)	Correlation between the virtual and real shoulder (T1) (*p-*value)	Correlation between the virtual and real shoulder (T2) (*p-*value)
Maxilla; incisors+canines (32)	0.53 (< 0.001)	0.45 (< 0.001)
Maxilla; premolars (57)	0.40 (< 0.001)	0.39 (< 0.001)
Maxilla; molars (75)	0.36 (< 0.001)	0.27 (< 0.001)
Mandible; incisors+canines (3)	0.89 (< 0.001)	0.89 (< 0.001)
Mandible; premolars (19)	0.27 (0.01)	0.43 (< 0.001)
Mandible; molars (71)	0.30 (< 0.001)	0.30 (< 0.001)
Maxilla; ø3.5 (110)	0.42 (< 0.001)	0.37 (< 0.001)
Maxilla; ø4.0 (6)	0.37 (0.06)	0.33 (0.105)
Maxilla; ø4.5 (40)	0.38 (< 0.001)	0.32 (< 0.001)
Maxilla; ø5.0 (8)	0.52 (0.001)	0.36 (0.038)
Mandible; ø3.5 (77)	0.31 (< 0.001)	0.34 (< 0.001)
Mandible; ø4.0 (3)	−0.05 (0.86)	0.53 (0.07)
Mandible; ø4.5 (10)	0.50 (< 0.001)	0.28 (0.07)
Mandible; ø5.0 (3)	0.00 (1.0)	0.26 (0.400)
ø3.5 incisors+canines (33)	0.53 (< 0.001)	0.45 (< 0.001)
ø4.0 incisors+canines (2)	0.79 (0.01)	0.83 (0.009)
ø4.5 incisors+canines (0)	–	–
ø5.0 incisors+canines (0)	–	–
ø3.5 premolars (70)	0.36 (< 0.001)	0.41 (< 0.001)
ø4.0 premolars (1)	−0.81 (0.18)	−0.31 (0.68)
ø4.5 premolars (4)	0.62 (0.01)	0.45 (0.07)
ø5.0 premolars (1)	0.27 (0.72)	0.54 (0.45)
ø3.5 M (84)	0.32 (< 0.001)	0.26 (< 0.001)
ø4.0 M (6)	0.09 (0.66)	0.40 (0.04)
ø4.5 M (27)	0.37 (< 0.001)	0.30 (< 0.001)
ø5.0 M (10)	0.34 (0.2)	0.29 (0.06)

**Table 3:** Changes over time between real shoulder positon in first (T1) and second (T2) examination

	*p-*value	T1 < T2 (%) Negative differences	T1 = T2 (%) No differences	T1 > T2 (%) Positive differences
Total	< 0.001	36.3	42.1	21.6
Maxilla	0.001	34.8	41.2	24.1
Mandible	< 0.001	39.0	43.8	17.2
Incisors+canines	0.001	38.6	45.7	15.7
Premolars	0.01	35.9	39.8	24.3
Molars	< 0.001	36.0	42.5	21.6
ø3.5	< 0.001	37.0	42.8	20.2
ø4.0	0.69	30.6	36.1	33.3
ø5.0	0.02	33.5	44.0	22.5
ø5.0	0.308	40.9	27.3	31.8

The position of the mucosal margin was stable in the esthetic anterior region in 45.7% of the cases. In 15.7% of the cases the vertical dimension of the peri-implant soft tissue was reduced, while increased in 38.6%. Across premolar and molar regions mucosa remained stable in 39.8 and 42.5% of the cases respectively, a reduction occurred in 24.3 and 21.6%, and an increase in 35.9 and 36% of cases respectively (Table 3).

An analysis of differences between the virtual and clinical position of the abutment shoulder showed mostly moderate positive correlation regardless of the implant platform diameter. However, when the results for each jaw were looked at separately, a strong positive correlation was noted for 5.0 mm diameter in the maxilla (*R* = 0.52) at T1. On the whole, the virtual shoulder vs. actual shoulder correlation was moderate (*R* = 0.36) at T2 (Table 2).

A parallel analysis at T1 (*R* = 0.53) for 3.5 mm diameter implants positioned in the anterior region revealed a strong positive correlation. Very strong correlation was present for 4.0 mm diameter implants placed in this area at both T1 and T2 (*R* = 0.79 and *R* = 0.83 respectively) (Table 2).

## Discussion

The aim of the current study was to evaluate whether a computer-planned submucosal position of the abutment shoulder would be in concord with its intraoral position after implant delivery and its functioning. The results demonstrated that there was a moderate correlation between the virtually planned and clinically measured position of the abutment shoulder. Clinical measurements were taken twice: first directly after the delivery of the abutment and temporary restoration (T1) and then prior to the cementation of the final crown (T2). A positive correlation was found to be moderate for implants in all positions at both clinical examinations. Moreover, a strong positive correlation was evident in all implants across the esthetic region (canines and incisors) at T1. At T2 an moderate positive correlation was found for all implants placed in the anterior esthetic regions (*R* = 0.46). This result is particularly significant as it affects the esthetic outcome in the anterior maxilla. Microanatomy of the soft tissue around implants may progressively change and differ from the tissue surrounding normal teeth. Disparities in blood supply and cellular attachment could be additional influencing factors [13, 14]. While osseointegration can be highly predicted, the response of the surrounding peri-implant mucosa is not clearly understood. Sources in the literature report mucosal recession in up to 16% of anterior single implants restorations. On the other hand, a spontaneous rebound of the receded soft tissue was recorded after a few years of functioning [15–18]. The literature does not seem to provide an objective assessment of the true cause of soft tissue instability. In an exhaustive systematic review Jung et al. [10] looked at the issue of peri-implant gingival recession both in terms of biological and esthetic outcomes. Interestingly, soft tissue complications, including dehiscences, occurred in 7.1% of cases after 5 years. In earlier studies the proportion was 9.7% after the same period [11]. In our study we found a reduction in tissue height in 21.6% of cases, an increase in 36.3% and no change in 42.1%. This is consistent with earlier studies and may indicate that rather than a chosen prosthetic technology, anatomical properties of peri-implant tissue should be blamed for the problem of soft tissue instability. Irrespective of a vertical increase or reduction in soft tissue, the study indicates unpredictability of a virtually planned abutment margin position. Confirmed in 80% of cases, soft tissue stability or growth, is a very positive point in favour of using computer-designed-manufactured abutments. However, soft tissue deficiency in over 21% of all cases poses a serious problem so to avoid such risk we suggests that the shoulder of a CAD/CAM abutment should be set slightly deeper submucosally than CAD software recommends. Naturally, when interpreting the results, in spite of our efforts and calibration of the examiner, there was a possibility of error due to the imperfection of the measuring technique. Limitations may arise from the fact that despite calibration, clinical measurement may be subject to human error as well as from rounding the measurement results to 0.5 mm. Predictable soft tissue stability in the current study can be explained by the adequate quality and quantity of peri-implant tissue, which did not require any grafting. According to a literature review by Basetti et al. [19], soft tissue condition should always be optimized prior to implant placement by all necessary grafting procedures, in order to achieve easier and more predictable treatment results. Soft tissue’s reaction to different surfaces of the abutment may explain its positive stability in the anterior zone (height reduction in 15.7% of cases). All abutments used in the anterior region were coated with titanium nitride at production (Atlantis™ Gold Hue), while those used in the premolar and molar region were made of pure titanium (Atlantis™ Titanium). Scarano et al. [20] described biocompatible properties of titanium nitride and showed that there was a significant reduction of bacterial count which can lessen the risk of inflammation within the peri-implant tissue. This may in turn stabilize soft tissue. A titanium nitride coating reduces bacterial load, diminishes its metabolic activity, adhesion and proliferation while it maintains biological affinity of TPS titanium surfaces towards bone cell precursors and promotes human gingival fibroblast adhesion [21–24]. Originally, the main reason for using titanium nitride coated abutments was esthetics, i. e. to minimize the grayish discoloration of marginal mucosa. But perhaps a more important purpose might be a greater soft tissue stability. Unfortunately, laboratory anodized abutments do not have such biocompatible characteristics as this technique creates pits, which intensify stress in the material and may progressively lead to micro-cracks in abutments and their failures [25–27].

Taking the above into consideration it is obvious that it is solely a clinician’s task to evaluate all risk factors, be it biological, biomechanical or esthetic, in each individual case, while respecting the patient’s preferences. As far as the danger of cement induced peri-implant tissue inflammation is concerned, a screw-retained restoration seems to be a better prosthetic solution [28, 29]. In some cases, however, due to technical limitations a cemented restoration seems to be preferable. In implants inserted in the posterior region, the abutment shoulder can be designed on a safe level for biological reasons. A problem arises in the front region, specifically when patients have high esthetic expectations. Their dissatisfaction may grow not only from development of gingival recessions, but also, as Benic and coworkers [30] revealed, from peri-implant mucosal discoloration visible at speaking distance, observed in 60% of implants. Inherently imperfect, currently available soft tissue transfer techniques seriously limit predictability of shoulders subgingival position. In both the analogue impression and optical scan, soft tissue always collapses towards the axis of the implant through lack of support provided by healing abutment or temporary restoration. The only way to keep precise characteristics of the emergence profile would be to superimpose a scanned image of the subgingival portion of the temporary crown onto the scanned image of the dental arch with the implant. This would significantly reduce a discrepancy between a digitally planned and actual intraoral position of the shoulder, but the technique is not yet commercially available [31–33].

## Conclusion

Within its limitations, this study concludes that the technique using a computer-designed position of the abutment shoulder in relation to the mucosal margin, can be predictably implemented in clinical practice. However, to avoid the risk of soft tissue deficiency, a clinician might consider setting the shoulder slightly deeper submucosally than CAD software routinely recommends. The results presented above need to be confirmed in further studies on larger groups.

## Abbreviations

CAD/CAM: Computer aided design/Computer aided manufacturing
T1: Examination after abutment delivery and temporization
T2: Examination prior to cementation of definitive crown
